# Innovative coaxial plastic stent within a lumen-apposing metal stent to prevent recurrence of pancreatic fluid collection

**DOI:** 10.1055/a-2629-0609

**Published:** 2025-08-28

**Authors:** Akihisa Kato, Michihiro Yoshida, Yusuke Kito, Tadashi Toyohara, Hidenori Sahashi, Yasuki Hori, Hiromi Kataoka

**Affiliations:** 1 Department of Gastroenterology and Metabolism, Nagoya City University Graduate School of Medical Sciences, Nagoya, Japan; 2 Department of Gastroenterology and Metabolism, Nagoya City University Graduate School of Medical Sciences, Nagoya, Japan; 3 Department of Gastroenterology and Metabolism, Nagoya City University Graduate School of Medical Sciences, Nagoya, Japan; 4 Department of Gastroenterology and Metabolism, Nagoya City University Graduate School of Medical Sciences, Nagoya, Japan; 5 Department of Gastroenterology and Metabolism, Nagoya City University Graduate School of Medical Sciences, Nagoya, Japan; 6 Department of Gastroenterology and Metabolism, Nagoya City University Graduate School of Medical Sciences, Nagoya, Japan; 7 Department of Gastroenterology and Metabolism, Nagoya City University Graduate School of Medical Sciences, Nagoya, Japan

Endoscopic ultrasound-guided transluminal drainage with a lumen-apposing metal stent (LAMS) is the favored initial approach to manage a symptomatic pancreatic fluid collection (PFC) 1 . The recurrence rate of PFCs was significantly lower when the LAMS was replaced with a double-pigtail plastic stent (DPPS), particularly for disconnected pancreatic duct syndrome 1 2 . However, replacing the LAMS with a DPPS can be challenging when the PFC cavity has shrunk, leaving insufficient space for the DPPS. The optimal strategy involves placing a coaxial DPPS within the LAMS concurrently during initial treatment and removing the LAMS afterward, leaving the DPPS in place to prevent PFC recurrence 3 4 . However, during LAMS removal, the DPPS may sometimes be unintentionally dislodged as well. To address this issue, we demonstrate an innovative coaxial plastic stent designed to facilitate the removal of only the LAMS while leaving the plastic stent in place.

A 43-year-old man was diagnosed with severe acute pancreatitis caused by alcohol consumption. Contrast-enhanced computed tomography revealed necrosis of the pancreatic body ( Fig. 1 **a** ). Although the pancreatitis resolved, an infected PFC developed due to disconnected pancreatic duct syndrome ( Fig. 1 **b** ). Endoscopic ultrasound-guided drainage was performed using a LAMS with an electrocautery-enhanced delivery system (Hot Axios; Boston Scientific, MA, USA) (fluid amylase, 93,185 IU/L). Concurrently, an innovative 5-Fr-diameter multi-loop, half-pigtail coaxial plastic stent (Gadelius Medical, Tokyo, Japan) was deployed within the LAMS ( Fig. 2 ). Two weeks after placement of the stents, the LAMS alone was removed, leaving the coaxial plastic stent in place. The removal procedure involved grasping the flange of the LAMS with forceps and passing the plastic stent through its lumen, with the proximal half-pigtail shape of the plastic stent preventing it from catching on the LAMS and ensuring it remained in place ( Fig. 3 , Video 1 ).

**Fig. 1:**
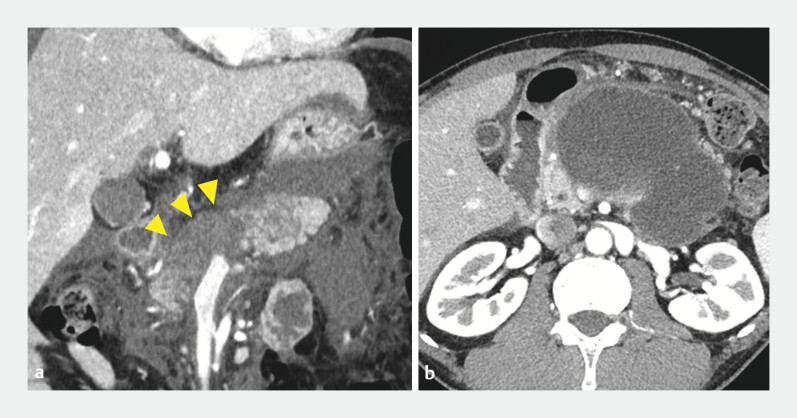
Contrast-enhanced computed tomographic images: **a** necrosis of the pancreatic body (arrowheads); **b** pancreatic fluid collection.

**Fig. 2:**
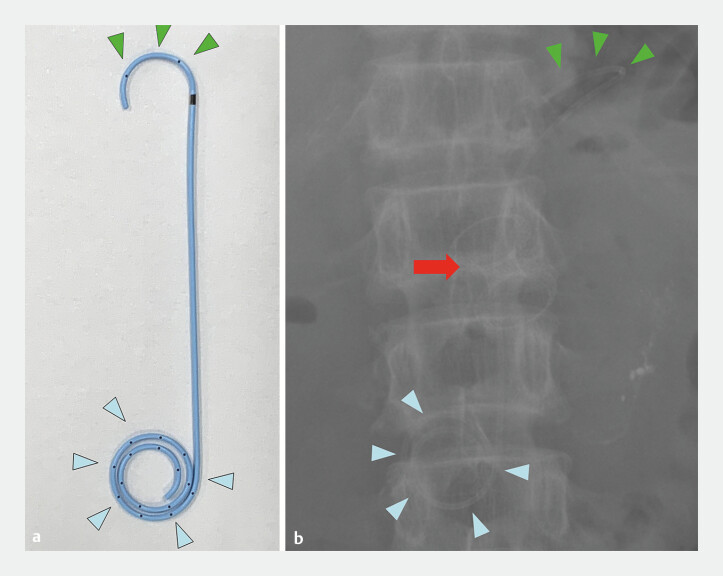
**a** The innovative plastic stent with a 5-Fr diameter multi-loop (blue arrowheads) and half-pigtail (green arrowheads). **b** Fluoroscopic X-ray image (red arrow: lumen-apposing metal stent, LAMS).

**Fig. 3:**
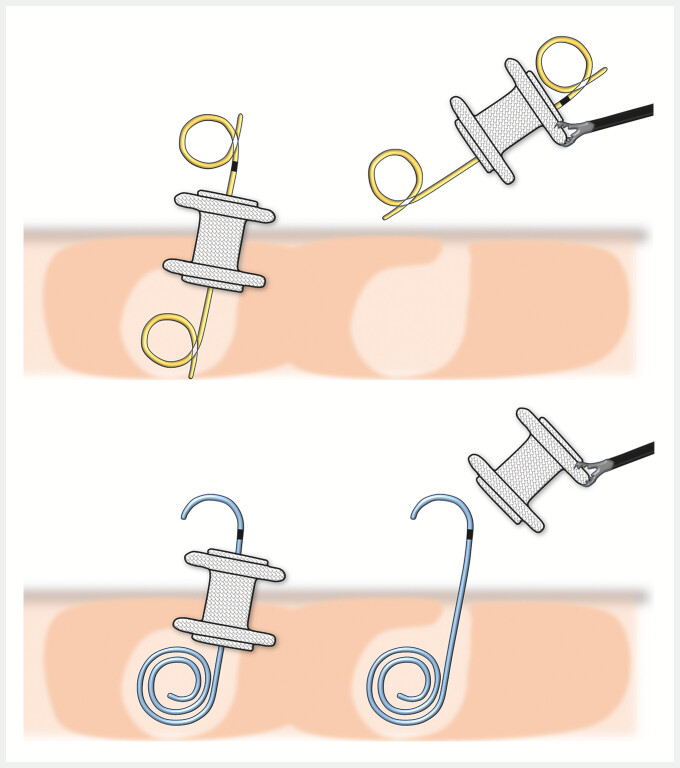
When a double-pigtail plastic stent (DPPS) has been placed coaxially within a LAMS during initial treatment, the DPPS may sometimes be unintentionally dislodged as well when the LAMS is removed. The proximal half-pigtail shape of the innovative plastic stent prevents it from catching on the LAMS and ensures it remains in place.

An innovative coaxial plastic stent facilitated the removal of the LAMS alone, leaving the plastic stent in place.Video 1

This innovative coaxial plastic stent within a LAMS may effectively prevent PFC recurrence, by remaining in place after LAMS removal.

Endoscopy_UCTN_Code_TTT_1AS_2AJ

Citation Format

Endoscopy 2025; 57: E353–E354. DOI: 10.1055/a-2587-8730

## Notes

Endoscopy E-Videos https://eref.thieme.de/e-videos
*E-Videos* is an open access online section of the journal *Endoscopy* , reporting on interesting cases and new techniques in gastroenterological endoscopy. All papers include a high-quality video and are published with a Creative Commons CC-BY license. Endoscopy E-Videos qualify for HINARI discounts and waivers and eligibility is automatically checked during the submission process. We grant 100% waivers to articles whose corresponding authors are based in Group A countries and 50% waivers to those who are based in Group B countries as classified by Research4Life (see: https://www.research4life.org/access/eligibility/ ). This section has its own submission website at https://mc.manuscriptcentral.com/e-videos .
